# Tocotrienols are good adjuvants for developing cancer vaccines

**DOI:** 10.1186/1471-2407-10-5

**Published:** 2010-01-06

**Authors:** Sitti Rahma Abdul Hafid, Ammu Kutty Radhakrishnan, Kalanithi Nesaretnam

**Affiliations:** 1 Malaysian Palm Oil Board, 6 Persiaran Institusi, Bandar Baru Bangi, 43000 Selangor, Malaysia; 2 Pathology Division, Faculty of Medicine and Health, International Medical University, 126 Jalan 19/155B, Bukit Jalil, 57000 Kuala Lumpur, Malaysia

## Abstract

**Background:**

Dendritic cells (DCs) have the potential for cancer immunotherapy due to their ability to process and present antigens to T-cells and also in stimulating immune responses. However, DC-based vaccines have only exhibited minimal effectiveness against established tumours in mice and humans. The use of appropriate adjuvant enhances the efficacy of DC based cancer vaccines in treating tumours.

**Methods:**

In this study we have used tocotrienol-rich fraction (TRF), a non-toxic natural compound, as an adjuvant to enhance the effectiveness of DC vaccines in treating mouse mammary cancers. In the mouse model, six-week-old female BALB/c mice were injected subcutaneously with DC and supplemented with oral TRF daily (DC+TRF) and DC pulsed with tumour lysate from 4T1 cells (DC+TL). Experimental mice were also injected with DC pulsed with tumour lysate and supplemented daily with oral TRF (DC+TL+TRF) while two groups of animal which were supplemented daily with carrier oil (control) and with TRF (TRF). After three times vaccination, mice were inoculated with 4T1 cells in the mammary breast pad to induce tumour.

**Results:**

Our study showed that TRF in combination with DC pulsed with tumour lysate (DC+TL+TRF) injected subcutaneously significantly inhibited the growth of 4T1 mammary tumour cells as compared to control group. Analysis of cytokines production from murine splenocytes showed significant increased productions of IFN-γ and IL-12 in experimental mice (DC+TL+TRF) compared to control, mice injected with DC without TRF, mice injected with DC pulsed with tumour lysate and mice supplemented with TRF alone. Higher numbers of cytotoxic T cells (CD8) and natural killer cells (NK) were observed in the peripheral blood of TRF adjuvanted DC pulsed tumour lysate mice.

**Conclusion:**

Our study show that TRF has the potential to be an adjuvant to augment DC based immunotherapy.

## Background

There are several types of vaccines used for prevention of infectious diseases, such as attenuated microorganisms, recombinant proteins and DNA vaccines. Currently, studies are being carried out in developing vaccines for tumours i.e. to activate the T-helper-1 (Th1) or cell-mediated arm of the immune system [1,2]. The cell-mediated arm of the immune system is crucial in protecting the host against the onset, development and spread of tumour. There are several ways to activate the cell-mediated arm of the immune system such as use of adjuvants, recombinant cytokines and nutritional supplements.

Breast carcinoma is the most common cancer in female and responsible for the largest numbers of cancer-related deaths in women. There are numerous studies looking at different approaches to inhibit or retard the growth of breast cancer in animal models and clinical trials [3-6]. One of these approaches involves the use of dendritic cells (DC), which are well-known for their potent ability as professional antigen-presenting cells (APC). These cells possess unique properties that allow these cells to elicit primary and boost secondary immune responses as well as to regulate the type of T cell- mediated immune response that is induced [7-9]. Several studies have demonstrated that tumour antigen-pulsed DC cells are capable of inducing the generation and proliferation of T-helper (Th) and cytotoxic T-lymphocytes (CTL) cells via by presenting tumour peptides on their major histocompatibility complex (MHC) class I and class II molecules respectively to mediate tumour immunity [1,10,11].

Nutrition is an important aspect to maintain a healthy and vigilant immune system. Hence, certain nutritional products or supplement may provide a boost for host immune system to fight tumour and this could improve the outcome of treatment. We have previously shown that vitamin E, namely the tocotrienol-rich fraction isolated from palm oil is effective in inhibiting the growth of human mammary cancer cell line in culture [5,12,13]. In this study, investigation on the effects of TRF supplementation, a non-toxic natural compound from palm oil to improve the efficacy of vaccines against breast cancer in our BALB/C mouse model has been carried out.

## Methods

### Mice

Inbred female BALB/c mice (six to eight week-old) were obtained from the Institute for Medical Research (IMR), Kuala Lumpur and housed at the Animal Maintenance Facility of the same institute. Animals were maintained on commercially available pellet diet and water *ad libitum*. The soda bedding was changed every three-to-four days. All experiments with animals were performed in accordance with the guidelines approved by the Ethics Committee of the Institute for Medical Research (IMR), Malaysia.

### Cell line

Murine 4T1 cell line, a spontaneously metastatic tumour cells derived from mammary gland tumour of BALB/c mice was purchased from the American Type Culture Collection (ATCC, Rockville USA). The 4T1 cells are comparable to human stage IV breast cancer [14]. These cells are poorly immunogenic and express surface MHC class I but not MHC class II molecules. The tumour cells were cultured in 25 ml cell culture flasks (Nunc, Denmark) as recommended by the ATCC.

### Medium and cytokines

Complete medium (CM) consisted of RPMI 1640 supplemented with 10% heat-inactivated foetal bovine serum, 1% Glutamine and 1% Penicillin/streptomycin. Recombinant mouse cytokines were purchased from Chemicon (USA) and used at the following concentrations: granulocyte macrophage colony-stimulating factor (GM-CSF) 10 ηg/ml; interleukin (IL)-4, 10 ηg/ml; recombinant mouse tumour necrosis factor (TNF)-α, 20 ηg/ml.

### Generation of bone marrow-derived DC

Murine bone marrow (BM) cells were harvested by flushing the marrow cavities of femur and tibia bones of six-to-eight week-old BALB/c mice with medium under aseptic condition. Erythrocyte-depleted mouse bone marrow cells were cultured in CM supplemented with GM-CSF and IL-4 [6,15] in a humidified 5% CO_2 _incubator (Heraeus, Germany). The medium was changed every three to four days. On day six, TNF-α was added to the DC cultures to induce maturation. The DC is harvested between day seven to nine. Cell differentiation was monitored by inverted microscopy. The expression of FITC-CD11c (BD PharMingen, USA), FE-mouse MHC class II and FITC-mouse CD86 (eBiosciences, USA) were analysed after seven to nine days of culture using a flow cytometer (FACS Callibur; BD Biosciences).

### Cytotoxic assay

The 4T1 cells, BM-derived DC and mouse splenocytes were plated at 1 × 10^5 ^cells/well in a microtitre plate and incubated at 37°C in a humidified 5% CO_2 _incubator (Heraeus, Germany) for 24 hours. The culture medium was replaced with fresh culture medium containing different concentrations (2 to 30 μg/ml) of TRF in 0.1% ethanol (final concentration v/v). Triplicate wells were used for each treatment. Control cells were incubated in complete RPMI 1640 medium containing just 0.1% ethanol (v/v). The cells were incubated at 37°C, 5% CO_2 _incubator (Heraeus, Germany) for 72 hours. After 72 hours, cell proliferation was determined using MTT cell proliferation assay according to the manufacturer's recommendations (Chemicon, USA). Percentage cell viability at different concentrations of TRF treatment was calculated based on the proliferation of the untreated control cells.

### Preparation of tumour cell lysate and antigen pulsing of DC

The 4T1 cells were cultured in the presence or absence on 8 μg/ml of TRF in T25 culture flasks overnight. The TRF-treated confluent 4T1 cells were harvested in a 15 ml tube (Falcon, USA). The cells were then resuspended in 1 ml complete medium. Tumour lysate (TL) was prepared by subjecting these cells three-to-five cycles of freezing in liquid nitrogen and thawing at 65°C. The cell lysates were spun at 2000 rpm for 5 min to remove particulate cellular debris. Untreated 4T1 cells were used as control.

### Inductions of IFN-γ and IL-12 released in DC culture *in vitro*

Freshly generated DC were plated at 1 × 10^6 ^cells/well in a 24-well plate overnight to allow the DC to adhere to the culture well. The medium was changed on the following day and 1 ml of tumour lysate preparation from TRF treated or untreated 4T1 cells were added to each well. After 72 h, the culture supernatants were collected. The amounts of IFN-γ and IL-12 in the culture supernatants were determined by a commercial ELISA according to manufacturer's recommendations (BD Biosciences, USA).

### Preparation of tumour lysate-pulsed DC for injection into mice

The freshly generated DC were incubated for 18 hours with tumour lysate from 4T1 cells and cultured in the presence or absence of TRF at a 1:1 ratio (DC:4T1). Following this, the TL-pulsed DCs were collected, washed three times with PBS, and resuspended in complete medium. Cell count was adjusted to 10 × 10^6 ^cells/ml in medium and this preparation was used for injection into mice.

### Ability of DC vaccinations and TRF supplementation to inhibit tumour growth in BALB/c mice

Five groups of five mice each were used for this study. The mice in DC+TL group were injected sub-cutaneously *(s.c) *in the left flank with 0.1 ml of 1 × 10^6 ^DC pulsed tumour lysate from 4T1 cells. The mice in DC+TRF group were injected sub-cutaneously *(s.c.) *in the left flank with 0.1 ml of 1 × 10^6 ^fresh DC. These mice were supplemented daily with oral TRF oil. The mice in DC+TL+TRF group were injected sub-cutaneously *(s.c.) *in the left flank with 0.1 ml of 1 × 10^6 ^DC pulsed tumour lysate and supplemented daily with TRF oil. The mice in TRF and control groups have not been treated with any DC injection but were supplemented with TRF and carrier oil daily. These injections were repeated on day 7 and 14. On day 28, the mice in all five groups were injected with a single *s.c. *containing 1 × 10^3 ^(0.05 ml) 4T1 cells in the right flank of their mammary breast pad. The mice were monitored daily for tumour growth and their body weight was recorded every week. Once tumour was palpable, the diameter of the tumour was measured using a calliper (Mitutoyo, Japan) as described previously [16]. After the mice were sacrificed, blood and spleen samples were collected for various analyses. The experiment was repeated twice.

### Analysis of whole blood by flow-cytometry

When the mice were sacrificed, blood was collected by heart puncture into heparinised tubes. The blood was stained with a number of monoclonal antibodies, including FITC-anti-mouse CD8α (BD PharMingen, USA) and FITC- Pan-NK cells-DX5 (BD PharMingen, USA) for flow-cytometry (FACSCallibur, Becton Dickinson, USA) analysis.

### IFN-γ and IL-12 secretion assay in splenocytes culture of experimental mice

For measurement of T-cell proliferation, the spleen was removed aseptically when the mice were sacrificed. The splenocytes (1 × 10^5 ^cells/well) were cultured for 72 h in culture medium supplemented with 10 μg/ml Concanavalin A (Con A) in a humidified CO_2 _incubator (Heraeus, Germany) at 37°C. After 72 h, the splenocyte culture was transferred into a sterile 1.5 ml tube and centrifuged (200 g × 5 min) to recover the culture supernatant. The amounts of IFN-γ and IL-12 in the culture supernatants were determined using a commercial ELISA kit (BD Biosciences, USA).

### Statistical analysis

The statistical significance of differential findings between experimental groups and control were determined using Student's *t *test. Significant values were indicated when two-tailed *P *values < 0.05.

## Results

### Characterisation of DC by using CD11c

**Figure 1:**
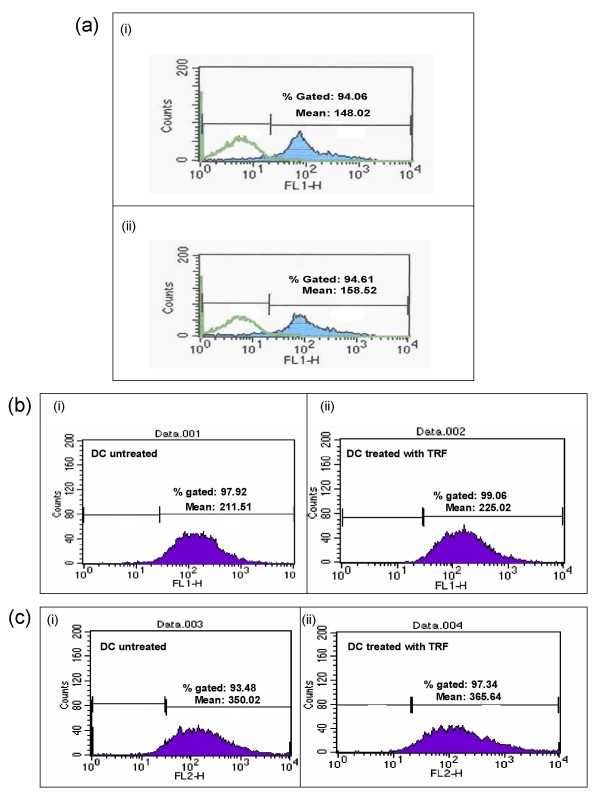
**(a) DCs stained with CD11c monoclonal antibody, i) untreated DC was stained with CD11c compared with unstained DC**. ii) Stained DC treated with 8 μg/ml of TRF compared with unstained DC. (b) DCs stained with CD86, i) untreated DC. ii) DC treated with 8 μg/ml of TRF. (c) DCs stained with MHC class II, (i) untreated DC. ii) DC treated with 8 μg/ml of TRF.

**Figure 2:**
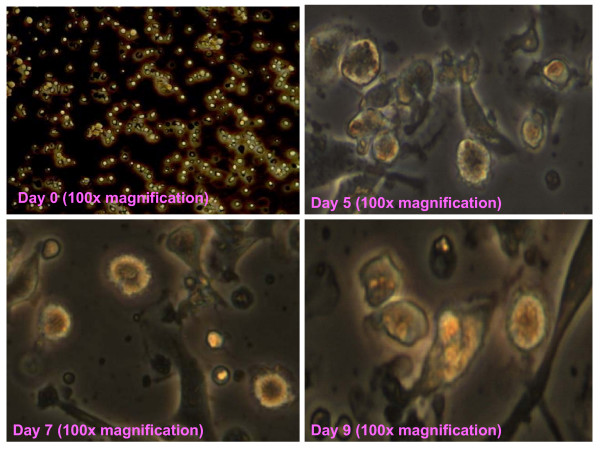
**Pictures of DC taken by microscopy with 100× magnification at day 0, 5, 7 and 9**.

The DC population was characterised using FITC-conjugated CD11c monoclonal antibody (BD PharMingen, USA). The results showed that more than 90% of DCs expressed CD11c in both TRF-treated and untreated DC after compared with unstained DC (see Fig. 1a (i) &1a (ii)). In Fig. 1b and 1c, the results showed that CD86 and MHC class II molecules also expressed more than 90% of DCs in both treated and untreated DC, but slightly higher in treated DC compared to untreated DC. The pictures of DC were taken by microscopy (Carl Zeiss, Germany) on day 0, 5, 7 and 9 are shown in Fig. 2. The dendrites were visible by day 5 and had multiplied by day 7 and 9. A similar growth pattern was observed for the size of the cells.

### TRF induces cell death in 4T1 tumour cells

**Figure 3:**
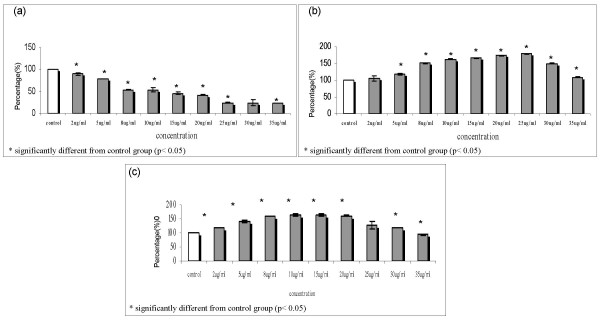
**Viability of (a) 4T1 cells decreased after treated with different concentrations of TRF**. IC50 recorded at 8 μg/ml of TRF treatment. b) Viability of DC increased in higher concentration of TRF up to 25 μg/ml. c) Splenocytes culture treated with different concentrations of TRF showed increased cell viability until 20 μg/ml of treatment.

The effect of TRF treatment on the viability of 4T1 cells, murine lymphocytes, and dendritic cells *in-vitro *after 72 h exposure to different concentrations of TRF was evaluated. The results show that TRF treatment inhibited the growth of 4T1 cells in a dose dependent manner (Fig. 3a). The inhibitory concentration, IC50 value of TRF for 4T1 cells was found to be 8 μg/ml. The viability of DC and splenocytes treated with TRF increased up to 25 μg/ml and (Fig. 3b) and 20 μg/ml (Fig. 3c) of TRF, respectively. Based on these findings, the concentration of TRF used in the preparation of tumour lysate from 4T1 cells for further studies was 8 μg/ml TRF as at this concentration it caused death in 50% of 4T1 cells but had no detrimental effect on the DC or splenocytes.

### IFN-γ an IL-12 released by DC after incubated with tumour lysate from 4T1

**Figure 4:**
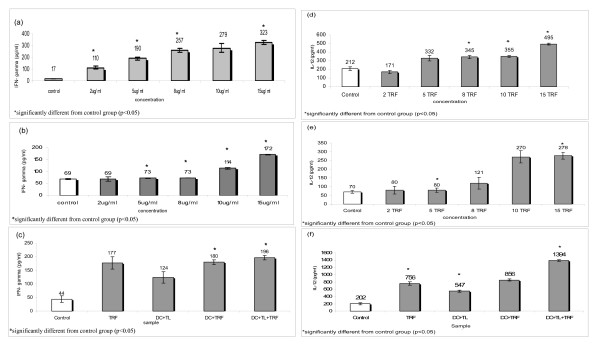
**The amount of IFN-γ produced by (a) DCs treated with different concentrations of TRF (2 to 15 μg/ml) and (b) splenocytes treated with different concentrations of TRF (2 to 15 μg/ml) (c) DCs co-cultured with tumour lysate from 4T1 cells in the presence of 8 μg/ml TRF for 72 h**. Following 72 hours of culture, the supernatants of control, TRF alone, DC with tumour lysate from 4T1 cells (DC+TL), DC with 8 ug/ml of TRF (DC+TRF), DC with tumour lysate from 4T1 cells and 8 μg/ml TRF (DC+TL+TRF) were harvested. The amount of IFN-γ in the supernatant was determined using ELISA. The amount of IL-12 produced by (d) DCs treated with different concentrations of TRF (2 to 15 μg/ml) and (e) splenocytes treated with different concentrations of TRF (2 to 15 μg/ml) (f) DCs co-culture with tumour lysate (DC+TL), in the presence of 8 ug/ml TRF (DC+TL+TRF), DC alone in the presence of ug/ml TRF (DC+TRF), TRF alone and control for 72 h.

Co-culturing of the DC with increasing concentrations of TRF (2 to 15 μg/ml) resulted in a dose-dependent increase in the production of IFN-γ by the DC (see Fig. 4a). Similar pattern for IFN-γ production was identified in splenocytes culture (see Fig. 4b). An increase in the production of IFN-γ was also observed with DC co-cultured with tumour lysate from 4T1 cells (see Fig. 4c). However, the production of IFN-γ was significantly (p < 0.05) augmented when 8 μg/ml TRF was added to this culture (see Fig. 4c). In Fig. 4d and 4e, the productions of IL-12 also increased with higher concentrations of TRF in DC and splenocytes cultures. Significant increase of IL-12 production was also detected in DC +TL+TRF treatment (see Fig. 4f).

### Pre-treatment with tumour lysate pulsed DC and TRF supplementation can inhibit tumour growth in BALB/c mice and enhance production of IFN-γ splenic leucocytes

**Figure 5:**
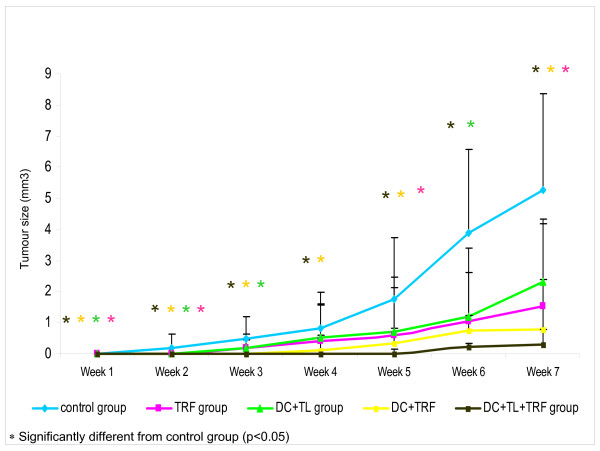
**Tumour size of the tumours induced in the control, TRF alone, DC+TL, DC+TRF and DC+TL+TRF groups were measured**. The DC+TL group were injected with DC that has been primed with tumour lysate from 4T1 cells and received daily oral supplementation of carrier oil while the DC+TL+TRF group were injected with DC primed with tumour lysate from 4T1 cells and supplemented daily with 1 mg/ml TRF orally. For the TRF alone, mice were only supplemented with TRF oil daily and for DC+TRF, DC fresh without tumour lysate and supplemented with TRF oil daily.

The effect of pre-treatment of mice with DC+TL+TRF to inhibit induction and growth of tumour was assessed using a mouse model of breast cancer. All the mice in the untreated control group rapidly developed tumour (see Fig 5, Table 1). Pre-treatment with tumour lysate pulsed DC (DC+TL) prior to the inoculation of 4T1 cells, significantly (p < 0.05) delayed tumour growth and size as compared to mice in the untreated control group (see Fig 5, Table 1). However, all the mice in the DC+TL group progressively developed tumours, albeit to a lesser size. In group supplemented with TRF alone (TRF group), the development of tumour was detected at week 3 and by week 6, the number of mice with tumour increase where three out of five mice (3/5) had tumour. At the end of the experiment, four mice in TRF group developed tumour but the size of the tumours was smaller compared to tumours developed by the mice in the DC+TL group. In the DC+TRF group, three out of five mice (3/5) developed tumour in the final week and the tumour growth remained static. In contrast, all the mice (5/5) treated with the combination of tumour lysate-pulsed DC pre-treatment and TRF supplementation (DC+TL+TRF) were protected against tumour growth up to week five (see Fig. 5, Table 1). However, two (2/5) of the mice in this group developed palpable tumours that remained static for several weeks. The remaining three (3/5) mice in this group remained tumour-free.

**Table 1:** The number of BALB/c mice developed tumour in each group.

Week after inoculation	Number developed tumour				
	
	Control (n = 5)	TRF (n = 5)	DC + TL (n = 5)	DC + TRF (n = 5)	DC + TL +TRF (n = 5)
1	0	0	0	0	0
2	3	0	0	0	0
3	5	1	1	0	0
4	5	2	2	2	0
5	5	3	3	3	0
6	5	3	5	3	2*
7**	5	4	5	3*	2*

* palpable and static

** end of experiment

**Figure 6:**
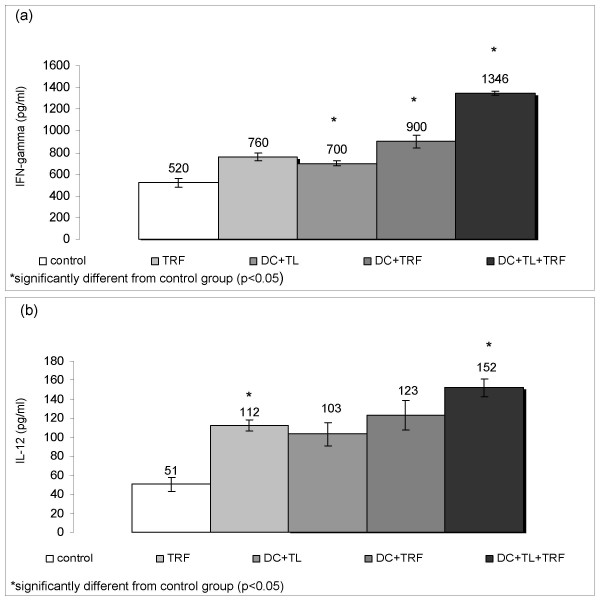
**Splenocytes (1 × 10^5 ^in each well) harvested from each group were cultured in the presence of 1 μg/ml Con A for 72 hours in 96 well plate**. The culture supernatant was collected and (a) IFN-γ produced was measured by using an ELISA kit. (b) IL-12 produced was measured by using an ELISA kit.

We also measured the amounts of IFN-γ and IL-12 produced by Con A-stimulated splenocytes from the control and treated mice (see Fig. 6a &6b). The splenocytes from mice that were treated with tumour lysate pulsed DC and daily supplementation of TRF (DC+TL+TRF) produced high amounts of IFN-γ (1346 pg/ml). The amount of IFN-γ produced by splenocytes cultured from the mice that only received tumour lysate pulsed DC (DC+TL) treatment and control were 700 pg/ml and 520 pg/ml, respectively. The splenocytes from the DC+TRF and TRF groups produced IFN- γ at levels of 760 pg/ml and 900 pg/ml. In general, IFN-γ produced by splenocytes from TRF supplemented mice was higher compared to non-TRF groups. For IL-12 productions, combination of tumour lysate pulsed DC with oral supplementation of TRF i.e. the (DC+TL+TRF) group produced higher amounts of IL-12 (152 pg/ml) compared to others (control = 51 pg/ml, TRF = 112 pg/ml, DC+TL = 103 pg/and DC+TRF = 123 pg/ml). These findings demonstrated that systemic administration of TRF could enhance the efficacy of tumour lysate pulsed DC immunisation *in vivo *and protective immunity to lethal tumour challenge.

### Immune regulation detected in the peripheral blood of treated mice groups

**Figure 7:**
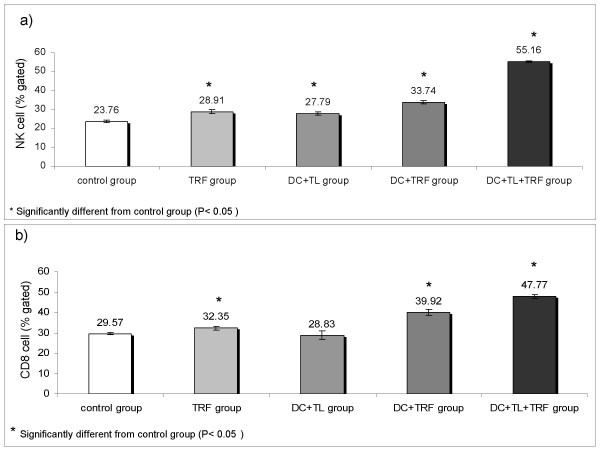
**The percentage of a) NK cells and b) CD8^+ ^T-lymphocytes in the control and experimental groups were determined by flow cytometric analyses**.

The percentages of natural killer (NK) cells and CD8^+ ^T-cells as assessed by flow cytometry are shown in Fig. 7. There was an apparent increased in the percentages of NK and CD8^+ ^T-cell in the DC+TL+TRF group. The date shows that immunisation of BALB/c mice with tumour lysate pulsed DC together with oral gavage of 1 mg TRF (DC+TL+TRF group) daily can increase the numbers of NK cells and CD8^+ ^T-cells, which could in turn lead to enhancement of the cell-mediated arm of the immune system that is crucial to fight tumours.

## Discussion

We have used a murine model of breast cancer where tumour growth in syngeneic female BALB/c mice is induced by a single *s.c. *injection of 1 × 10^3 ^4T1 cells in the right flank of the mice. The 4T1 tumour model closely resembles human breast cancer because its poor immunogenicity and ability to spontaneously metastasise to lungs, liver, bone marrow and brain [16,17]. This model was used to evaluate the efficacy of oral supplementation of TRF to enhance the therapeutic benefits of DC immunotherapy in treating breast cancer in a mouse model.

Our results showed that TRF on its own, in both the *in-vitro *or *in-vivo *methods used in this study can significantly inhibit the growth of 4T1 cells or tumours induced by these cells. This study corroborates with previous reports of other tumour models [12,18,19]. In this study, we show for the first time, that the combination of using two therapeutic approaches i.e. three injections of tumour-lysate pulsed DC prior to inoculation of tumour cells and daily TRF supplementation can significantly inhibit the growth of tumour as well as improve the overall survival of mice induced with tumour.

The MTT assay showed that 2 μg/ml TRF could inhibit the proliferation of 4T1 tumour cells *in vitro*. However, TRF at this concentration did not exhibit any anti-proliferative effect of DC or murine splenocytes (Fig. 3b &3c). The IC_50 _value of TRF for 4T1 cells was determined to be 8 μg/ml. Although there was a slight increase in the CD11c expression by TRF-treated DCs compared to control, this increase was not statistically significant. The DC+TL+TRF treatment yielded significantly higher production of IFN-γ by the DC. In addition, the DC pulsed with tumour lysate from 4T1 cells could enhance the productions of IFN-γ and IL-12 by T-helper-1 cells when these cells were cultured in the presence of 8 μg/ml TRF. These findings show that TRF is a potent compound that can induce the immune system to release cytokines that promote cell mediated immune response.

In the animal model, mice that were injected with DC pulsed with tumour lysate from 4T1 cells and supplemented daily with oral TRF showed marked reduction in tumour onset and growth. Previous reports by Nesaretnam and co-workers [12,13,19-21] have shown that tocotrienol on its own can inhibit growth of human breast cancer cells *in vitro *[12,19] as well as in athymic nude mice [21]. The combination of tumour lysate pulsed-DC and TRF supplementation observed in this study could inhibit the growth of breast tumour in the mouse model. As shown in Fig. 5 and Table 1, the incidence of tumour in the DC+TL+TRF group showed smaller tumour burden compared to control group and DC+TL group, TRF group and DC+TRF group. The splenocytes from the DC+TL+TRF group produced the highest amount of IFN-γ (1346 pg/ml) compared to the DC+TL group (700 pg/ml), control untreated group (520 pg/ml), TRF group (760 pg/ml) or DC+TRF group (900 pg/ml). The IL-12 amount produced in splenocytes culture from experimental mice showed similar pattern as IFN-γ. There was a significant higher production of IFN-γ and IL-12, which are the two signature cytokines for Th1 response, by splenocytes from the DC+TL+TRF group which also suggest that the combination therapy has been effective in enhancing a cell-mediated immune response in these mice. Interferon-γ also promotes class-switching to IgG isotype, which is a key component of cell mediated immunity [22]. This cytokine also upregulates the expression of class II MHC molecules and B7 co-stimulatory molecules on antigen presenting cells. For IL-12, it is expressed specifically in macrophages and dendritic cells [17,23] and plays a central role in mediating cell mediated immunity, promoting differentiation of CD4+ T cells to the Th1 subset and of CD8^+ ^T cells into mature cytotoxic T lymphocytes (CTLs) [24]. IL-12 is also a potent stimulator of NK cells as well as enhancing cytocidal anti tumour immune responses [25,26]. All these actions could serve to amplify T-cell responses [22]. Our findings are in agreement with that reported by Ramanathapuram and co-workers [2], who showed that combination therapy using alpha-tocopheryl succinate (α-TOS) and DCs (α-TOS+DC) increased IFN-γ production by CD4^+ ^and CD8^+ ^T lymphocytes. The α-TOS is an esterified analogue of vitamin E used as an adjuvant to demonstrate the inhibitions of 3LL tumours *in vitro *and in C57BL/6 mice model. In our study, we used tocotrienol-rich fraction (TRF), which contains the natural isomers of vitamin E. The TRF can be found abundantly in palm oil was used as an adjuvant to develop cancer vaccine in this study because TRF could inhibit the growth of murine mammary cancer (4T1) and human breast cancer (MCF-7 & MDA) cells. In addition, when the DC were co-cultured with TRF, there was a significant increase in the production of IFN-γ by the DC. These are important properties for an adjuvant to be used in a tumour model to have as it could promote Th1 immune responses.

Both NK cells and CD8^+ ^T-cells have a crucial role in the recognition and removal of tumour cells [27-29]. The NK cell activation induced by tumour cells can directly promote anti-tumour responses. Although activation of CD8^+ ^T-cells is more complex, it is important for the development of tumour-specific memory T-cells, which is responsible for long-term protection against the same tumour [28]. The percentages of NK cells and CD8^+ ^T-cells increased in the TRF supplemented group compared to the control and DC+TL only group. Thus, the combine therapy of using DC+TL and daily oral TRF supplementation can promote tumour-specific immune responses in this mouse model of breast cancer.

## Conclusion

In conclusion, this study reports on the ability of TRF to act as an adjuvant that promotes tumour-specific cell-mediated immune response in mice. The daily TRF supplementation augments the protective effect provided by three vaccinations of DC pulsed with tumour lysate from 4T1 cells in this mouse model. Further studies are needed to investigate in detail the effectiveness of TRF adjuvanted DC immunotherapy.

## Abbreviations

4T1 cell: Mouse mammary cancer cell; APC: antigen-presenting cells; ATCC: American Type Cell Culture Collection; BM: bone marrow; CD8: cluster of differentiation 8; CD11c: cluster of differentiation 11c; CM: complete medium; CO_2_: carbon dioxide; Con A: concanavalin A; CTL: cytotoxic T-lymphocyte; DC: Dendritic Cell; ELISA: Enzyme Linked Immuno Sorbent Assay; FITC: Fluorescein isothiocyanate; GM-CSF: granulocyte macrophage colony-stimulating factor; IFN-γ: Iinterferon-gamma; IL-4: interleukin 4; IL-12: interleukin 12; MHC: major histocompatibility complex; NK cell: natural killer cell; *s.c*: sub-cutaneously; Th-1: T-helper-1; TL: Tumour lysate; TNF: tumour necrosis factor; TRF: tocotrienol-rich fraction.

## Competing interests

The author(s) declare that they have no competing interests. This project was funded by a research grant from the Malaysian Palm Oil Board (project number PD46/04) and the International Medical University (project number IMU 142-2007).

## Authors' contributions

SRAH carried out the whole research and drafted the manuscript; AKR and KN conceived the study and participated in its design coordination and helped to draft the manuscript. All authors read and approved the manuscript.

## Pre-publication history

The pre-publication history for this paper can be accessed here:

http://www.biomedcentral.com/1471-2407/10/5/prepub
